# Family life and autistic children with sensory processing differences: A qualitative evidence synthesis of occupational participation

**DOI:** 10.3389/fpsyg.2022.940478

**Published:** 2022-10-20

**Authors:** Gina Daly, Jeanne Jackson, Helen Lynch

**Affiliations:** Department of Occupational Science and Occupational Therapy, University College Cork, Cork, Ireland

**Keywords:** meta-ethnography, meaningful participation, occupation, family-centered practice, autistic children, sensory processing, routines

## Abstract

Autistic children with sensory processing differences successfully navigate and engage in meaningful family daily occupations within home and community environments through the support of their family. To date however, much of the research on autistic children with sensory processing differences, has primarily been deficit focused, while much of the caregiver research has focused on issues of distress, burden, effort, and emotional trauma in coping with their child's diagnosis. This study aimed to conduct a qualitative evidence synthesis, using a meta-ethnographic approach to explore the gap identified in understanding successful occupational experiences of family participation and daily family routines when supporting an autistic child with sensory processing differences and to offer an alternative strengths-based perspective. Inclusion criteria were studies which were peer-reviewed qualitative design, published from 2000 to 2021, and that concerned parents/caregivers' perspectives of family occupations of children diagnosed with autism spectrum disorder. Studies were electronically searched in eight databases from October to December 2021 and 23 studies were identified which met the inclusion criteria. Noblit and Hare's seven step approach for conducting analysis in meta-ethnography was used, and three themes identified: (1) sensory processing differences in daily life, (2) what is hard about hard, and (3) orchestrating family life. Results identified the centrality of sensory experiences in understanding family life. Living with unpredictability while orchestrating certainty through routines was core to successful participation. This review provides insights into how parents negotiate the complexities of constructing family life when living with an autistic child. The results can inform the design of future interventions that specifically address the relationship between meaningful participation in family occupations and daily routines and sensory processing in autistic children.

**Systematic review registration:**

https://www.crd.york.ac.uk/prospero/display_record.php?ID=CRD42022298938, identifier: CRD42022298938.

## Introduction

The World Health Organization (2022) states that the global incidence of Autism Spectrum Disorder (ASD) is 1% and therefore it is the most prevalent neurodevelopmental disorder in childhood. As a neurodevelopmental disorder, ASD is still largely understood *via* the medical or deficit model. For example, ASD is diagnosed when there is evidence of particular behaviors or communication skills that differ from typically developing children (American Psychiatric Association, 2013). Core features in such a diagnosis include (a) persistent deficits in social communication and social interaction across contexts, not accounted for by general developmental delays, (b) restricted, repetitive patterns of behavior, interests, or activities (c) symptoms must be present in early childhood and (d) symptoms together limit and impair everyday functioning (American Psychiatric Association, 2013). In addition, in 2013, the APA included atypical sensory reactivity (over or under responsive) as a further ASD criterion (Robertson and Simmons, 2013; Tavassoli et al., 2014), which, until then, had long gone unrecognized. Indeed, studies have found that 80–90% of autistic children^1^ experience significant difficulties in sensory processing which influences their participation in daily activities (Lane et al., 2010; Lloyd et al., 2013; Williams et al., 2018). Yet, it is the impact of these symptoms on social participation, and on education, employment and wellbeing that is a most significant concern for families of autistic children, and the potential risk of poverty of experience, and ultimately occupational deprivation (Durocher et al., 2014; Wilcock and Hocking, 2015).

The challenge in enabling social participation is complex for autistic children and their families, and for the services who work with them. It requires an integrated understanding of how the core symptoms of ASD combine to influence and steer the child to develop and experience meaningful daily occupations, in the context of their social and physical environments. When exploring meaningful occupations for autistic children and their families this translates to understanding how a child's sensory differences are embodied within their daily occupational experiences. A child's intolerance for dressing may be due to the feel of certain clothing, reactivity to the taste and smell of certain foods could result in the restriction of many foods based on their sensory properties, the need for increased vestibular input for sensory regulation may require regular visits to the playground and attending the local shopping center during peak opening times could escalate a child's auditory hyperreactivity. Within this context, there has been an increased exploration within the field of behavioral science to understand how sensory experiences influence brain-behavior relationships within the autistic population (Wolff et al., 2012), Studies of autistic children who have sensory processing differences show that they integrate sensory information differently to typical children, and present with sensory differences across different senses (Kern et al., 2008; Schoen et al., 2009; Lane et al., 2010; Marco et al., 2011). For example, studies have demonstrated a marked difference between autistic children and typically developing children regarding their tactile defensiveness and lower tolerance to tactile stimuli (Baranek et al., 2006; Tomcheck and Dunn, 2007). These difficulties have been found to include atypical responses to textures, an abnormal detection of tactile stimuli (Blakemore et al., 2006) preoccupations with sensory features of objects, and problems habituating to prior sensory experiences (Tannan et al., 2008). So, evidence exists that sensory differences are significantly associated with the core features of ASD (Lane et al., 2014; Zachor and Ben-Itzchak, 2014) and within this evidence, sensory reactivity is the most discussed and acknowledged sensory processing difference and as such is the primary focus of this research (Botha et al., 2021).

As noted earlier, such sensory differences among autistic children impacts on the nature of their participation in daily life. Autistic children may have different needs in being able to participate in activities of daily living at home (White et al., 2007; Schaaf et al., 2011), particularly where a child has sensory over-responsivity or reactivity (Reynolds and Lane, 2008). Sensory reactivity can significantly influence everyday functioning in occupations (Bagby et al., 2012; Reynolds et al., 2012; Bodison, 2015). Indeed, studies have found a significant relationship between sensory reactivity and occupational performance in activities of daily living for autistic children, including sleep, dressing, eating, engaging in play and participation in leisure and school related activities (Miller Kuhaneck and Britner, 2013; Mazurek and Petroski, 2015). However, sensory processing differences influence not only the lives of autistic children but also the context within which they live. Consequently, families of autistic children have also been the focus of significant study across cultures, to understand how families experience living with ASD, including experiences of the diagnostic process (Khara et al., 2021), of marginalization (Chiaraluce, 2018), pathologicalization of ASD (Mackay and Parry, 2015), adjusting and coping with life with an autistic child (Kapp and Brown, 2011; Harrop et al., 2018), parental identity and stress (Rocque, 2010) and how it impacts parental quality of life (Fong et al., 2021; Beheshti et al., 2022). Overall, these studies all address the significant impact of living with an autistic child and tend to prioritize the subsequent limitations that result on family participation in work, family, and leisure activities. While these studies provide insight into family life, they primarily examine parental experiences of difficulties, and of living an arduous life, from a deficit perspective, which has been highlighted in other studies (Boyd et al., 2014).

In order to understand successful, meaningful participation in family life, one place to start is to explore how parents structure family life which for autistic children typically involves the use of routines (Boyd et al., 2014). The adoption of routines in family life is typically associated with transmission of family and cultural values, as well as providing structure to family occupations (Boyce et al., 1983; Spagnola and Fiese, 2007). For families of autistic children, predictability within their daily life is an important feature (Boyd et al., 2014). However, this means that families are required to structure their family routines around the autistic child, to remove spontaneity, and avoid unplanned family events (Boyd et al., 2014). In this way, routines can be considered a double-edged sword, whereby there is a cost to family values in order to benefit the child, which Larson describes as a paradox (Larson, 1998). Yet for these families, routines crucially provide stability to what can be a frightening world (Boyd et al., 2014), and have been found to promote healthier coping mechanisms among families of autistic children (Kapp and Brown, 2011). Further exploration of the role of routines in family life with older autistic children is less well known however, and warrants further study (Boyd et al., 2014).

From this preliminary review of evidence, it is clear that living with an autistic child presents challenges, yet there is an inadequate understanding beyond the deficits and difficulties, of what works well in daily life and what shared participation within the home environment might look like for families with autistic children. While evidence has been previously synthesized relating to routines specifically (e.g., Boyd et al., 2014), to our knowledge no study has been conducted to date that synthesizes evidence for composing meaningful family life more generally. Thus, the purpose of this study was to analyze multiple studies of parental perspectives, views and experiences in parenting an autistic child with sensory processing differences and synthesize the means by which they have successfully negotiated challenges and effectively supported autistic children within their families. The aims of the study were to strengthen our understanding of meaningful family occupation by exploring: (1) What is known about parental perspectives of autistic children and sensory processing differences within the context of family life and routines (2) How do families overcome the challenges that their child experiences to co-construct daily routines and occupations within their home environment, and (3) How do parents and their children optimize meaningful engagement in family occupations. This evidence has the potential to inform intervention and service delivery through generating new understandings of the experiences of parents, and their autistic children within the family context and the wider family unit, in order to more effectively meet parents needs relating to successful family participation (Anaby et al., 2014).

## Methods

### Design

This qualitative synthesis used a meta-ethnographic approach as detailed by Noblit and Hare (1988) and follows the eMERGe guidelines in reporting the synthesis, which is recommended when reporting meta-ethnographies in particular (France et al., 2019). Meta-ethnography is one of the most consistently used approaches to qualitative evidence synthesis in healthcare (Cahill et al., 2018) because of its effective and robust methods of strengthening the evidence through synthesis. Meta-ethnography offers a well-delineated approach to the synthesis of qualitative research which produces novel interpretations and conceptual innovation of the area of interest. This approach was chosen by the authors as it provided a method to examine and reinterpret the current evidence base in a new and novel way, producing innovative findings to inform the field of practice. Subsequently, a preliminary search of the literature indicated that there were enough studies to merit a meta-ethnography. A study protocol for this meta-ethnography was registered and published on Prospero | (Registration number: CRD42022298938) (Daly et al., 2022).

### Search strategy

Initially the search was a pre-planned comprehensive search to seek all available studies. The search strategy then became iterative to prioritize theoretical sampling (Booth, 2016; Cahill et al., 2018). The search strategy was developed initially from reviewing qualitative literature on parental perspectives of children with autism spectrum disorder and sensory processing. Support was then received from an academic librarian in University College Cork, Ireland. A combination of keywords, thesaurus and MeSH terms were utilized. Keywords used in the search were drawn from recently conducted systematic reviews for autistic children and from a review on strategy searching for qualitative research. The search strategy combined three concepts which were central to the research objective (see Table 1).

**Table 1:** Search strategy terms.

• autism OR autism spectrum disorder* OR autistic spectrum disorder* OR ASD OR asperger* OR HFA
• “parent* perspective*” OR “caregiv* perspective*” OR famil*
• “sensory processing*” OR “sensory processing dysfunction” “sensory integration*” OR SPD* OR “sensory integration difficulties”
• Qualitative OR mixed methods
• “family routines” OR “occupational participation” OR “activities of daily living” OR “family life” OR “occupational engagement”

The SPIDER search strategy tool (sample, phenomenon of interest, design, evaluation, research type) was used to structure the process for screening and the selection of studies as it is identified as a more effective tool compared to the more traditional PICO approach (Methley et al., 2014; Booth, 2016). A systematic search of peer-reviewed studies was conducted in September 2021 using eight databases from health, science, education, and humanities to ensure the inclusion of diverse perspectives: Academic Search Complete, CINAHL, ERIC, MEDLINE, PsycINFO, Scopus, Web of Science and PubMed. Searches were limited to English language publications between the dates 2000–2021, so as to capture the most recent research in the field. The PRISMA-checklist for systematic reviews was used to illustrate the search strategy procedures.

### Inclusion/exclusion criteria

Primary research studies using only qualitative methods of data collection and analysis to explore parental perspectives of the occupational participation of autistic children and young persons (3–18 years) with sensory processing differences in daily life were included. All cultural and geographic contexts were considered and settings such as home and the community where the parent is present with the child were included. Studies were excluded if (a) they employed mixed methods or where only a quantitative design was employed, (b) had a co-occurring physical disability and/or whose child did not have a diagnosis of autism spectrum disorder. In addition, if the studies primary focus data was not on the child's daily routines and participation in family occupations (for example, studies in airports, school, or dentist), they were excluded.

### Screening

Once duplicates were removed, the first author (GD) and a second reviewer (e.g., HL or JJ) screened all titles and abstracts against the defined eligibility criteria. Each paper was screened by two reviewers to check for consistency and rigor. Subsequently, full-text review for all eligible papers was conducted by two reviewers. Each reviewer independently considered the paper's relevance to this qualitative synthesis. Ambiguities were addressed ***via*** a third reviewer to resolve differences of opinion. The entire screening process is presented ***via*** a PRISMA flowchart.

### Data extraction and data synthesis

The synthesis was conducted using the seven phases of meta-ethnography originally described by Noblit and Hare (1988). The seven phases are as follows: (1) Getting started, (2) Deciding what is relevant to the initial interest, (3) Reading the studies, (4) Determining how the studies are related, (5) Translating the studies into one another, (6) Synthesizing translations, and (7) Expressing the synthesis. In contrast to other forms of systematic reviews, in meta-ethnography, theoretical sampling is used to identify studies that provide rich data rather than including every study identified (Atkins et al., 2008; Cahill et al., 2018). The analysis aims to create third-order constructs or themes from first order constructs (respondents' quotations) and second-order constructs (authors' interpretation). Each of the included full-text studies were imported into NVivo qualitative data analysis software to facilitate extraction of second-order concepts, coding and comparison. As suggested, by Noblit and Hare (1988), all studies were read several times in full. Key quotations, metaphors, and concepts related to parental perspectives of daily routines and family occupations in autistic children were extracted using the words and explanations provided by the authors (second-order constructs). Throughout the process of meta-ethnographic analysis and synthesis, two reviewers completed initial coding and data extraction independently and collaborated and compared findings regarding emerging themes. Studies were translated into each other, and a reciprocal translation was conducted for this synthesis, as the studies concerned similar concepts (Noblit and Hare, 1988; Toye et al., 2014).

### Quality appraisal

Two reviewers independently appraised each of the 23 papers included in the review. The quality of the included studies was assessed using the Critical Appraisal Skills Programme (CASP) checklist (Critical Appraisal Skills Programme, 2022). The CASP is a checklist specifically designed for the formal appraisal of qualitative research and was chosen as it provides a systematic process to identify the strengths and weaknesses of a research study. Each item was recorded as “Yes”, “No”, “Unclear” or” Not applicable”. Once complete, the appraisal findings were contrasted, variations in decisions were examined and consensus was reached ***via*** discussion between both reviewers (HL and GD) and when required with the third reviewer (JJ). We made a decision in advance not to exclude studies with low quality scores, as the focus of the review was around conceptually rich data on autistic children and families and their occupational participation. Quality appraisal meetings between the team were conducted fortnightly whereby each of the studies was scrutinized using the well-defined inclusion/exclusion criteria.

## Results

### Study selection

**Figure 1:**
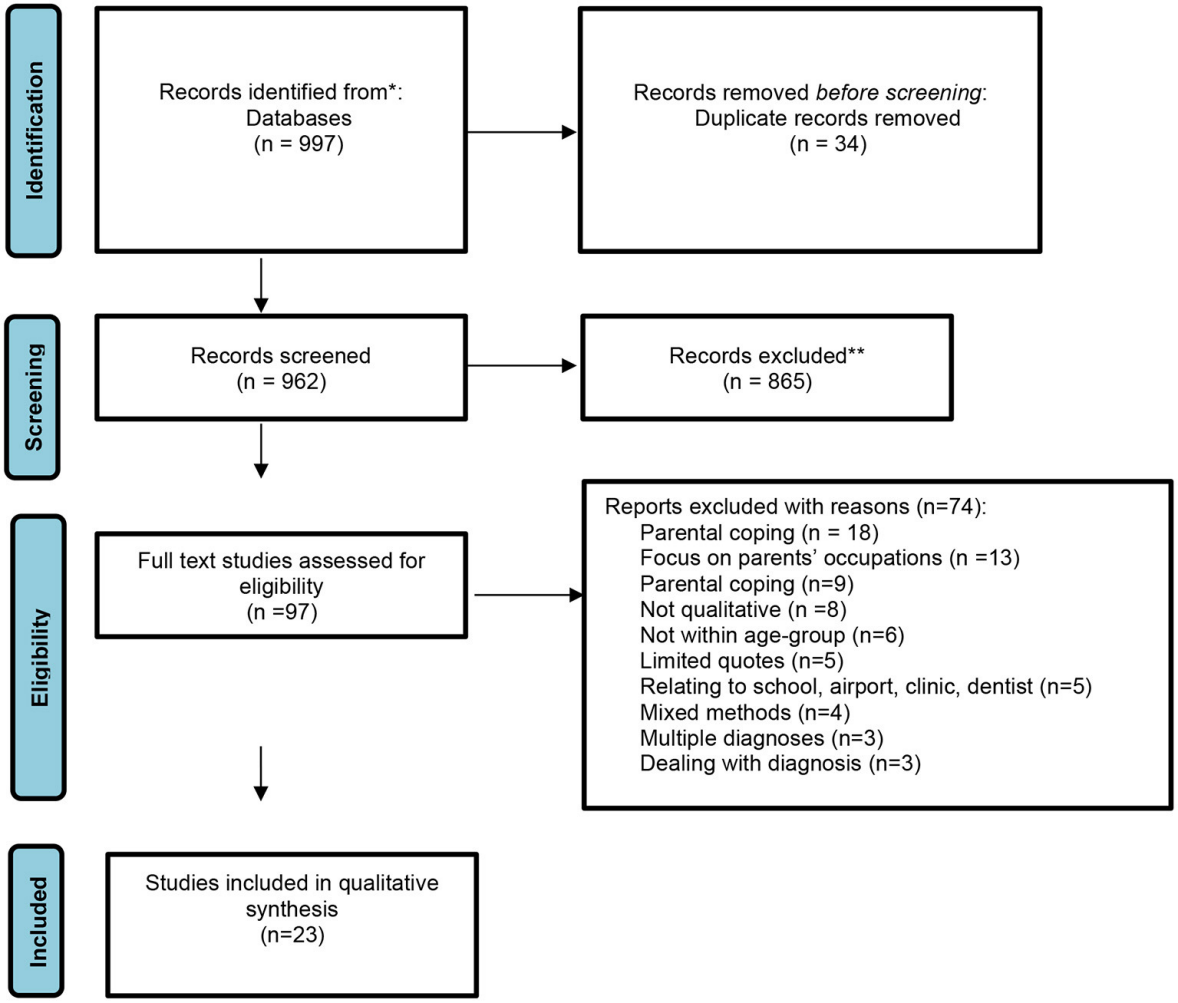
Adapted PRISMA flow diagram of study selection process (Moher et al., 2009).

Initial searches yielded 997 results, 963 after removing duplicates prior to screening. Screening by title and abstract excluded 865 studies, leaving 97 studies for full text review. Seventy-four studies were excluded and 23 met eligibility and were included in the review (November 2021). Figure 1 presents a PRISMA Flowchart diagram, detailing the entire process, which led to the inclusion of 23 studies. The 23 studies are represented by numbers to support the flow and readability of the synthesis section.^2^

### Study characteristics

Twenty-three papers were identified for synthesis from this search and are listed here alphabetically (Burkett et al., 2021; Burrows et al., 2008; Columna et al., 2020; DeGrace, 2004; DeGrace et al., 2014; Dickie et al., 2009; Epstein et al., 2019; Galbraith and Lancaster, 2020; Harwood et al., 2019; Keller et al., 2014; Kim et al., 2018; Kirkpatrick et al., 2019; Larson, 2006, 2010; Marquenie et al., 2011; Naik and Vajaratkar, 2019; Potter, 2017; Redquest et al., 2020; Rios and Scharoun Benson, 2020; Schaaf et al., 2011; Shannon et al., 2021; Suarez et al., 2014; Tokatly Latzer et al., 2021) (see text footnote 2).

A detailed summary of all aspects of the 23 included articles from the study is provided in Table 2. The majority of identified articles were from USA (*N* = 11), with other represented countries including Canada (*N* = 4) Australia (*N* = 4), UK (*N* = 1), India (*N* = 1), Israel (*N* = 1) and Ireland (*N* = 1). Most studies used interviews to collect data (DeGrace, 2004; Larson, 2006, 2010; Dickie et al., 2009; Marquenie et al., 2011; Schaaf et al., 2011; DeGrace et al., 2014; Keller et al., 2014; Suarez et al., 2014; Potter, 2017; Kim et al., 2018; Epstein et al., 2019; Harwood et al., 2019; Naik and Vajaratkar, 2019; Columna et al., 2020; Redquest et al., 2020; Rios and Scharoun Benson, 2020; Shannon et al., 2021; Tokatly Latzer et al., 2021). The remaining studies used focus groups (Kirkpatrick et al., 2019; Burkett et al., 2021); Qualitative ethology (Burrows et al., 2008); and photovoice (Galbraith and Lancaster, 2020). A combined total of 301 parents/caregivers/families of autistic children aged between (3–18 years) were included across the studies, with study sample sizes ranging from three to 37 parents/caregivers. Of these, the majority (95%) of the studies included parents of primary school aged children within their sample, with 26% of studies including parents of secondary school aged children. Parents of children aged between 16 and 18 years were represented in 13% of studies.

**Table 2:** Article characteristics.

**Article number**	**References Country**	**Title**	**Methods as described in the study**	**Participants**	**Country and ethnicity**	**Focus of study relating to participation in family occupations**
1	Burkett et al. (2021) USA	Restricted eating in pre-schoolers with Autism: Mother stressors and solutions	Focus group and ethno-nursing design	11 mothers of pre-school children (3–6 years)	9 = non-Hispanic/Caucasian from a large Midwestern city 1= African American 1= Asian American.	Mealtimes routines and preferences
2	Burrows et al. (2008) Canada	Sentinels of safety: Service dogs ensure safety and enhance freedom and well-being for families with autistic children	Participant observation and video; semi-structured interviews	10 families (children 4.5–14 years)	Southwestern Ontario (Canada)	Family activities in the home and public outings
3	Columna et al. (2020) USA	The experiences of Hispanic families of children with autism spectrum disorder regarding physical activity	Semi-structured telephone interviews	9 parents (Hispanic families) (children 6–14 years)	Hispanic Parents - Participants resided in five different states in the U.S. (Georgia, Florida, New York, Massachusetts, and Texas) and one participant did not report their state of residence.	Recreational activities, exercise and hobbies
4	DeGrace (2004) USA	The everyday occupation of families with children with autism	In-depth interviewing	5 families (5 children 9–10 years)	USA	Everyday occupations of families
5	DeGrace et al. (2014) USA	Families' experiences and occupations following the diagnosis of autism	Semi-structured interviews	7 families (7 children 3–18 years)	USA	Family occupations
6	Dickie et al. (2009) USA	Parent reports of sensory experiences of preschool children with and without autism: a qualitative study	Telephone or face-to-face interviews	Parents of 66 pre-schoolers (37 parents of autistic children 6–17 years))	USA (White, Black and Hispanic included)	Responses to sensory experiences (food-related, self-care)
7	Epstein et al. (2019) Australia	Parent-observed thematic data on quality of life in children with autism spectrum disorder	Semi-structured interviews	21 parents (19 mothers, 2 fathers) (children 6–17 years)	Parents living in Australia. Country of Birth for Parents: Australia (10) and other (11) which included Argentina, England, Scotland, Germany, Poland, Ireland, New Zealand, Singapore	Relaxation, natural environment, routines and social connection
8	Galbraith and Lancaster (2020) Australia	Children with autism in wild nature: Exploring Australian parent perceptions using photovoice	Photovoice	3 Participants (children 5–10)	Australia	Nature and the outdoors, and balancing needs of sibling
9	Harwood et al. (2019) Australia	Parental perceptions of the nature of the relationship children with Autism Spectrum Disorders share with their canine companion	Case design - interviews	11 mothers (children aged 5–12)	Western Australia	Companionship and influence of assistant dog on sensory experiences relating to sleep and social connection
10	Keller et al. (2014) USA	Relationships of children with Autism Spectrum Disorders and their fathers	Semi-structured interviews	7 fathers (children 4–6 years)	USA	Shared family activities, fathering
11	Kim et al. (2018) USA	Listening to the screaming whisper: a voice of mother caregivers of children with autistic spectrum disorder (ASD)	Semi-structured interviews	12 mothers (average age of child was 9)	Indiana, USA	Leisure and recreation: negotiation and constraint
12	Kirkpatrick et al. (2019) Ireland	Qualitative study on parents' perspectives of the familial impact of living with a child with autism spectrum disorder who experiences insomnia	Focus groups	15 parents (15 children 4–12 years)	Ireland	Bedtime routine, eating, sleep, social connections
13	Larson (2006) USA	Caregiving and autism: how does children's propensity for routinization influence participation in family activities?	Semi-structured interviews	9 participants (children 3–14 years)	USA based (6 Caucasians of European descent, 1 Puerto Rican/African-American, 1 Chinese, and 1 Mexican)	Routines in family life including restaurant, leisure activities, morning routines
14	Larson (2010) USA	Ever vigilant: Maternal support of participation in daily life for boys with autism	Semi-structured interviews	9 mothers (children 3–8 years)	USA based varied in self-identified ethnicity (6 Caucasians of European descent, 1 Puerto Rican/African-American, 1 Chinese, and 1 Mexican),	Routines in self-care, leisure and social activities
15	Marquenie et al. (2011) Australia	Dinnertime and bedtime routines and rituals in families with a young child with an autism spectrum disorder	Semi-structured interviews	14 mothers (children 2–5 years)	Australia	Routines: bedtime and dinnertime
16	Naik and Vajaratkar (2019) India	Understanding parent's difficulties in executing activities of daily living of children with Autism Spectrum Disorder	Semi-structured interviews	20 participants (fathers = 9 and mothers = 11) (children 5–9 years)	India	Self-care activities including eating, toileting, dressing, brushing, grooming, sleep
17	Potter (2017) UK	Fathers experiences of sleeping problems in children with autism	Semi-structured interviews	25 fathers (20 children: 15 were under 10 years)	Fathers living in the UK (24 white and 1 Black)	Sleep challenges, and fathers management of night-time waking
18	Redquest et al. (2020) Canada	Social and motor skills of children and youth with autism from the perspectives of caregivers	Semi-structured interviews	8 participants (children 6-16 years)	Canada	Physical hobbies, social skills concerning physical activity
19	Rios and Scharoun Benson (2020) Canada	Exploring caregiver perspectives of social and motor skills in children with Autism Spectrum Disorder and the impact on participation	Semi-structured interviews	17 participants (mothers, fathers and 1 grandmother) (children 5–9 years)	Canada	Participation in social activities and influence of motor skills and social skills
20	Schaaf et al. (2011) USA	The everyday routines of families of children with autism Examining the impact of sensory processing difficulties on the family	Semi-structured interviews	4 Families (children 7–12 years)	USA white, non-Hispanic	Participation in family activities inside and outside the home
21	Shannon et al. (2021) Canada	“There's nothing here”: Perspectives from rural parents promoting safe active recreation for children living with autism spectrum disorders	Open ended & semi-structured interviews	12 parents (10 mothers, 2 fathers) of children (3–12 years)	Canada	Participation in safe active recreation in rural areas
22	Suarez et al. (2014) USA	Phenomenological examination of the mealtime experience for mothers of children with autism and food selectivity	Semi-structured interviews	4 mothers (children 6–9 years)	USA - 3 white non-Hispanic and 1 white Hispanic	Mealtime routines and preferences
23	Tokatly Latzer et al. (2021) Israel	Core experiences of parents of children with autism during the COVID-19 pandemic lockdown	Semi-structured interviews	Parents of 25 children (children 4–6 years)	Israel - low (*n* = 7), middle (*n* = 8), and high (*n* = 10) socioeconomic backgrounds recruited	Adjusting to home routines during COVID-19 lockdown

### Quality appraisal

All 23 studies were of high quality based on criteria used in meta-ethnographies as they all received “yes” answers for at least 7–10 of 10 CASP checklist questions (see Table 3).

**Table 3:** CASP qualitative research scoring tool (rated as yes [green], no [red], unclear [purple]).

**References**	**Question 1: Was there a clear statement of the aims of the research?**	**Question 2: Is a qualitative Methodology appropriate?**	**Question 3: Was the research design appropriate to address the aims of the research?**	**Question 4: Was the recruitment strategy appropriate to the aims of the research?**	**Question 5: Was the data collected in a way that addressed the research issue?**	**Question 6: Has the relationship between researcher and participants been adequately considered?**	**Question 7: Have ethical issues been taken into consideration?**	**Question 8: Was the data analysis sufficiently rigorous?**	**Question 9: Is there a clear statement of findings?**	**Question 10: How valuableis the research?**
Burkett et al. (2021)	Yes	Yes	Yes	Yes	Yes	Yes	Yes	Yes	Yes	
Burrows et al. (2008)	Yes	Yes	Yes	Unclear	Yes	No	Yes	Yes	Yes	
Columna et al. (2020)	Yes	Unclear	Yes	Yes	Yes	Yes	Yes	Yes	Yes	
DeGrace (2004)	Yes	Yes	Yes	Yes	Yes	Yes	Yes	Yes	Yes	
DeGrace et al. (2014)	Yes	Yes	Unclear	Yes	Yes	No	Yes	Yes	Yes	
Dickie et al. (2009)	Yes	Yes	Yes	Yes	Yes	No	Yes	Yes	Yes	
Epstein et al. (2019)	Yes	Yes	Yes	Yes	Yes	No	Yes	Yes	Yes	
Galbraith and Lancaster (2020)	Yes	Yes	Yes	Yes	Yes	No	Yes	Yes	Yes	
Harwood et al. (2019)	Yes	Yes	Yes	Yes	Yes	No	Yes	Yes	Yes	
Keller et al. (2014)	Yes	Yes	Yes	Yes	Yes	Yes	Yes	Yes	Yes	
Kim et al. (2018)	Yes	Yes	Unclear	Yes	Yes	No	Yes	Yes	Yes	
Kirkpatrick et al. (2019)	Yes	Yes	Yes	Yes	Yes	Yes	Yes	Yes	Yes	
Larson (2006)	Yes	Yes	Yes	Unclear	Yes	No	Yes	Yes	Yes	
Larson (2010)	Yes	Yes	Yes	Unclear	Yes	No	Yes	Yes	Yes	
Marquenie et al. (2011)	Yes	Yes	Yes	Yes	Yes	No	Unclear	Yes	Yes	
Naik and Vajaratkar (2019)	Yes	Yes	Unclear	Unclear	Yes	No	Yes	Yes	Yes	
Potter (2017)	Yes	Yes	Yes	Yes	Yes	NO	Unclear	Unclear	Yes	
Redquest et al. (2020)	Yes	Yes	Yes	Yes	Yes	No	Unclear	Yes	Yes	
Rios and Scharoun Benson (2020)	Yes	Yes	Yes	Yes	Yes	Yes	Unclear	Yes	Yes	
Schaaf et al. (2011)	Yes	Yes	Yes	Yes	Yes	No	Unclear	Yes	Yes	
Shannon et al. (2021)	Yes	Yes	Yes	Yes	Yes	No	Yes	Yes	Yes	
Suarez et al. (2014)	Yes	Yes	Yes	Unclear	Yes	No	Unclear	Yes	Yes	
Tokatly Latzer et al. (2021)	Yes	Yes	Yes	Unclear	Yes	No	No	Yes	Yes	

### Synthesis

This meta-ethnographic synthesis of qualitative data synthesized first order and second order constructs from the 23 studies which resulted in the identification of three core themes (third order constructs): (1) Sensory differences and routines in daily occupations, (2) What is hard about hard, and (3) Orchestrating family life. Table 4 presents the 23 studies and how they contributed to the themes and subthemes.

**Table 4:** Contribution of included studies toward themes.

	**References**	**Theme 1: sensory differences**			**Theme 2: what is**		**Theme 3: orchestrating**	
		**and routines in daily occupations**			**hard about hard?**		**family life**	
		**Occupational experiences in sensory worlds**	**Forensic sense making of sensory experiences**	**Routines in daily occupations of families**	**The hard work in establishing routines**	**The relentless need for vigilance**	**Positive sensory experiences for the child**	**Doing family differently**
1.	Burkett et al. (2021)		X		X			
2.	Burrows et al. (2008)			X	X	X		X
3.	Columna et al. (2020)				X	X		X
4.	DeGrace (2004)			X		X		X
5.	DeGrace et al. (2014)			X		X	X	
6.	Dickie et al. (2009)	X	X	X	X		X	X
7.	Epstein et al. (2019)	X	X		X		X	X
8.	Galbraith and Lancaster (2020)	X	X				X	X
9.	Harwood et al. (2019)	X					X	
10.	Keller et al. (2014)			X	X			
11.	Kim et al. (2018)						X	X
12.	Kirkpatrick et al. (2019)		X	X	X			X
13.	Larson (2006)			X	X	X		X
14.	Larson (2010)	X		X	X	X		X
15.	Marquenie et al. (2011)			X	X			
16.	Naik and Vajaratkar (2019)	X		X	X		X	
17.	Potter (2017)			X	X			
18.	Redquest et al. (2020)	X					X	
19.	Rios and Scharoun Benson (2020)				X			
20.	Schaaf et al. (2011)	X	X	X	X			
21.	Shannon et al. (2021)				X			X
22.	Suarez et al. (2014)		X	X	X			
23.	Tokatly Latzer et al. (2021)		X	X	X			X

### Theme 1: Sensory differences and routines in daily occupations

The first theme relates to how parents experience living with a child with sensory differences. Three subthemes were identified: occupational experiences in sensory worlds, forensic sense making of sensory experiences, which allowed parents to understand these processes further and routines in daily occupations of families.

#### Occupational experiences in sensory worlds

Parents described a multitude of occupational experiences that can be understood from a sensory perspective, primarily relating to auditory and tactile sensitivity. Auditory sensitivity was a common theme spoken about from the parents' perspective, and parents reported on the severe impact these sensory experiences were having on their children and how they impacted their daily occupations and family routines: “*It's not just the loudness. It's the intensity. He perceives it so clearly that he goes into the moment. He can't separate himself from it*” (14). Children frequently responded by “*having a meltdown”* due to unexpected unpredictable sudden sounds, or from too many competing sounds such as fire alarms, toilet flushes in public restrooms, dogs barking, other children crying, loud coughing (6, 8). Auditory sensitivities were particularly evident in family outings to museums, movies, amusement parks, or religious events which were often accompanied by sensory qualities, such as unexpected loud noises (14). However, even ordinary occupational routines such as vacuuming was discussed by many as upsetting and distressing their child (6, 7, 9, 18, 20). “*She's slightly sensitive toward noise …. if there is a lot of chaos going on she does become really quite agitated, and they don't help”* (14).

Parents reported on the tactile experiences of their children and how this altered the bathing occupations of their child. “*I get him out of the bathtub and wrap him really tight in the towel. I do it quick.... If you start wiping him instead of wrapping him in a towel to try and get the water off... that is something that is aversive to him”* (20). Children often experienced distress from self-care occupations requiring tactile input, relating to their face and head (6), such as having their ears cleaned, having their face touched, and having haircuts. One mother reflected on her child's experiences: “*I'm not sure if it is exactly painful or not. But it's definite he feels it differently than we do, that's for sure”* (6). Occupational experiences of dressing were also documented and associated with tactile sensitivity: “*He does not like tight fitting clothes and clothes with tags”* (16). Consequently, these children avoided wearing certain types of cloth materials, printed clothes, and clothes with tags and collars (16). Overall, parents expressed the realization that their child experienced senses differently, that this experience was real, and even perhaps painful, and certainly caused distress (6, 8). This is indicative of how parents have a unique and invaluable insight into their child's lived experience within their daily occupations.

#### Forensic sense making of sensory experiences

Forensic sense-making of sensory experiences was a recurring concept across these papers and conveys the need to conduct constant scientific analysis and interpretation of physical evidence, in order to understand what the child's sensory experiences were. This second sub theme relates to how parents engaged in an ongoing process of detective-work, and that this could be confusing, and required a forensic approach: “*What's the issue? How can we help them? because I don't get it. You know, I have been with this kid for 8 years. And, uh, I still don't get it”* (22). Many parents reflected on the erratic and unpredictable pattern to their child's sensory processing needs. One mother was particularly mystified by her child's sensory needs: “*My mind is constantly on... What can I do now? How can I handle this? [he's telling me] the car seat... It's not firm enough... it's like a sensory integration thing... I'm tired of thinking*” (12). Parents tried to make sense of their children's responses to sound “*Maybe his reactions are just a little brisker than most people...”* (6). Being able to understand what sounds bother a child, under what circumstances, makes it possible for parents to avoid situations, prepare the child, or use other strategies to diminish the impact on the child. “*I have no idea why he likes things. I don't know if he's experiencing it in the same way I would*” (6). Yet, parents showed an intense understanding of sensory influences because of this forensic work: for example, where the child avoided the vacuum cleaner only when it was turned on but was seen to play with it when it was turned off proved to this family that the child was sensitive to the loud sound and not the object (6).

Parents often hypothesized why their child liked various sensations “*she loves being under water. Maybe the pressure of the water, the blocking out maybe of certain sounds?”* (7). Unusual sensory experiences presented puzzles that parents tried to understand “*why you would need to jump up and down, you know, and make yourself feel good, or, you know, why you constantly need to chew on stuff”* (6). One parent reported that after her child engaged in swimming activities, he would have to have a P-chewy device: “*We have got to have a P-chewy right there and he needs like a minute or two [of chewing]. I don't know if it is because of all the input of the water and swimming that he just needs to kind of download*...” (20).

Forensic sense-making existed concurrently with confusion. For example, in relation to food sensitivities one parent said: “*Could it be the flavor, could it be the color, could it be the sensory aspect, could it be this, could it be constipation? It's over analyzing things… to the point of exhaustion. It's like you have to cover so many bases for one simple problem”* (1). Parents put themselves in the child's shoes and reflected “*I don't know how I'd go eating something that was different to what I expected*” (8). There appeared to be confusion over mealtimes in that one strategy may work 1 day but not on another: “*He KNOWS the difference. He refuses; he will just spit it out unless it's exactly right. Like, even macaroni and cheese. If I cook the noodles for 2 min too long and they get soggy, he won't touch it. Even though it's the same exact ingredients*” (22). Parents detailed understanding of their child's interoceptive cues was also discussed across the papers and was evident in relation to the child's variable hunger responses: “*He is always saying, I'm hungry, I'm hungry, especially at bedtime. I sometimes think he is hungry … and then … is he getting enough to eat but you just don't know”* (12). “*All day long he opens the refrigerator. He just wants to eat all day. He can't get full. He just stuffs more and more things in his mouth and he cries and shouts that he wants more food. He's getting fat and it's unhealthy”* (23). Parents knowledge, attitudes and practices of their child's individual physical health needs was a prominent feature and demonstrated the essential resource they have in managing their child's success in daily occupations.

#### Routines in daily occupations of families

Routines as a way of living life were a significant theme in these studies and highly valued as a means to mitigate the sensory-emotional world experienced by the child. Functioning routines were proposed as the main way to order and structure life and integrate the child into family occupations across childhood (4, 13). Indeed, the purpose of routines went beyond this and served to provide reassurance to the autistic child, that once a routine was in place the child “*knows that all is well with the world”* (13), and without routines, the child could not cope: “*it would be awful without some kind of routine at night, he would have a meltdown, he just couldn't cope without a routine*” (12). Overall, routines helped the child in a number of ways, by providing predictability and clarity therefore of expectation, to manage transitions more easily and to reduce anxiety and thus develop confidence in themselves (6, 13). They consequently provided parents with comfort in knowing the child was secure and able to participate and enjoy family life (2, 4, 14).

Routines involved a predetermined set of steps *within a task* like bathing (e.g., undressing, playtime in the bath, washing, drying), or *within the event* like preparing for bed (e.g., teeth cleaning, toileting, dressing for bed, story reading) (15, 23). Routines were also embedded in temporal contexts with set times for getting up or going to bed each day (16). While all studies explored daily occupations in general, some papers focused intensively on mealtime and bedtime routines that are consistently documented as most challenging for families of autistic children (12, 15, 16, 17). For mealtimes, for example, one study documented the diverse influences on how a child might react at mealtimes when “*issues related to food were not limited to one sensory aspect but rather included texture, taste, smell, visual aspects of the food itself, and having the food on hands or tongue.”*(6). This awareness of sensory influences warranted a lot of thought and planning to ensure that the sensory experiences related to mealtimes accounted for the child's needs, and therefore were predictable and avoidant of novelty. This frequently involved multiple meals being cooked for all family members (22). For many families, mealtimes were rarely a time for togetherness emotionally or physically.

For the daily routine of bedtime, there was a core ritual of performance required: families described it in this order: “*the sequence of routines tended to involve: bathing, teeth cleaning, toileting, dressing in pajamas; then play/television or story reading; good night hugs/kiss, having a drink, getting a comfort toy, followed by lights out and lying down in bed with the child to assist transition to sleep”* (15). For bedtime routines, parents used their knowledge of the sensory sensitivities to devise sensory calming techniques to assist with settling the child to sleep, which included extra blankets, soothers or pacifiers, and low lighting (15). For some families the assistant dog provided the extra comfort for the child, enabling more successful sleep not just for the child but the parents also (2).

Common across the studies was the experience of anxiety in these children around bedtime. “*He would be fairly hyper in the evening time before getting to bed, so that it would impact on everybody. No-one gets any peace to do things”* (12). Although many families worked hard to establish bedtime routines that were predictable and calming for the child, nonetheless, children continued for many years to experience anxiety at bedtime and had extreme difficulties with sleep resulting in sleep deprived families (12, 17). This was often related to anxieties about the next day: “*If there's something happening at school that he wasn't happy about like going on a trip or something, you know out of the ordinary, he wouldn't like that. So, he would be worrying about it and he wouldn't sleep”* (12). Some families resorted to co-sleeping as a result (12, 17) but this family routine also became disruptive for the marital relationship: “*The fact that he is almost nine and still sleeping with me and you know my husband is working so he sleeps in another room. I struggle with that because it's making our relationship strained”* (12). Parents reflected on how the autistic child's sleep routine had to match the whole families “*My child will not go to sleep unless everybody in the house goes to sleep”* (16). In this instance, families were shown to be actively problem solving methods of interconnecting the child and families sleep needs, to allow for overall improved sleep for the family.

Routines were a way of enfolding family occupations into daily life and as such allowed the family to function. For example, one study (14) talked of how family members were able to find personal time for their preferred occupations once the autistic child was engaging independently in their own routines, demonstrating the positive effect of routines in family life. Yet for many, family occupations needed to be done in such a way that allowed for rapid adjustment, depending on the responses of the autistic child and determined by their sensory needs (20). This demonstrates how fluid and adaptable these families are in their ability to weave their child's needs into their daily life. A shared sense of joy was evident when everyday routines went well such as having a kiss and a hug before bed (10), parent–child hugging or snuggling routine (6), sitting in a restaurant when the child is content during the meal (5), touching or lying beside their assistant dog (2), when the child performed a new skill for the first time (jumped) (10). Overall, the outcome of orchestrating predicable and functioning routines was to achieve a “*reasonable life for family members*” (13).

### Theme 2: What is hard about hard?

The second theme of “what is hard about hard” consisted of two subthemes: The hard work in establishing routines and the relentless need for vigilance, which reflects the backdrop to constructing family life. Parents documented what exactly was ‘hard about hard' and how new ways of parenting were therefore required within this theme.

#### The hard work in establishing routines

Considerable skill, resilience and efficiency were required to develop routines (13, 14, 23). For example, families noted that although a child might engage in a routine, it often took a lot longer than expected to complete it which added much frustration in family life (13, 23). Some families talked of routines being impossible to implement or maintain however (12, 16, 17). This was often associated with the ever changing sensory and emotional needs of the child that were often difficult to identify as noted earlier (6) which one author described as the “wild card in daily routines” (13). One study (13) described it as “*a dance between creating a structure and then improvising depending on the child's responses, while minimizing the child's need to change in instances of anxiety*.” A key feature across these studies therefore was the need to ‘*pick your battles'* as a way of constructing family life (15).

Building on the forensic sense making of sensory experiences from theme one, was the consensus that the design of routines required consistent “detective work” (14) and consistently involved consideration for the physical and sensory environment which determined the choice of tools for daily occupations, such as cups, plates, toothbrushes (1, 13), and for some was enhanced by the presence of an assistant dog (2). Designing routines also involved an understanding of how the child learns best and might include the use of verbal instruction (23) or visual schedules (16). Common to many studies was that functioning routines take significant time to develop in getting the child to try new activities and form new habits (1, 3, 19, 22). However, the outcome when a child achieved some new skill or routine was identified as extra special as a result (10).

Daily occupations were imbued with a high level of vigilance, due to the child's occupational behavior for example, roaming the house at night (5) or elopement and getting lost outdoors (21). Consequently, for daily occupations parents talked of needing to constantly build and orchestrate routines by drawing from a range of strategies: ordering, sequencing, predicting, restructuring, accommodating, performing. Sensory sensitivities commonly governed daily routines, and parents strove to understand the complex intersensory experiences of their children, for example, knowing the child's oral sensitivities for eating (6) or knowing to avoid tight clothes or clothes tags (16). Doing the small sequences of an occupation in the same order every day was a significant goal for some families (20).

Routines had a specific role and for some, family life did not require routines to be in place 100% of the time. Routines worked best when they provided an overall structure, with predictable patterns of activity (12). They also required flexibility, (7) with some families talking of needing a lack of structure at home to provide space for the child to unwind after school and place no demands on him: “*My son is calmer and quieter now, because no one is demanding anything from him. At school there are many demands, and there is discipline. At home it's much gentler and much more flexible*” (23). This difference between expectations at home and school diminished during COVID pandemic when lockdown resulted in many families dealing with home-schooling and dealing with the reality of the pandemic: “*My son had several events of anger outbursts during the night. He was wild and crazy. He wouldn't go back to sleep and screamed. I turned to a sleep clinic but due to the situation they are not working*” (23).

#### The relentless need for vigilance

While families within these studies explained processes for establishing functional routines, the child's inability to tolerate change in routine, the sensory environment and daily life was fundamental to how family life was hard. The natural consequence was an extreme commitment to developing routines to counter this inability to cope with change, and the “*all-encompassing extreme vigilance”* that was therefore required to support the child to take part in family life (14). As with all caregiving duties of young children, vigilance, safety and managing risks is to be expected. But the level of vigilance described in the studies reviewed, captured a more intense hardship, from the parents' perspective.

Relentless vigilance can be described as the moment to moment on guard approach taken by parents to ensure their children were in a manageable state to engage in occupations, this too included managing their child's sensory regulation in any given situation. Perhaps most consuming for parents was the anticipatory vigilance as expressed in one study “*There is this underlying current of “it's about to happen, he's going to start spiting*” (4). This persistent experience of “*somethings going to happen*” was repeatedly stated by caregivers as exhausting. “*We're all emotionally tired. We're all physically tired. We don't know if he's going to flip out if we go to somebody's house or... if somebody comes to our house... Even if he doesn't it's like a lot of work to... keep him even”* (14). Another study also reported that parents find vigilance permeates everything and as a result “*nothing we do is ever easy relaxing is difficult”* (14). Heightened sensory sensitivities of the child which were commonly associated with heightened emotional responses, led many parents to engage in hypervigilance. This often included living a life of high anxiety (6, 7, 12).

Constantly being on duty was a core feature to what was hard about hard and the impact on the family was immense: “*Your whole family's life is always revolving around this situation, making compromises, because of doing extra work... he makes all the basic things a lot more harder, whether you're having a meal, whether you're taking care of your everyday activities, it's a lot of work”* (4). These compromises often revolved around the child's sensory preferences and needs (6, 9, 19, 20). This control of events was because the child was unable to cope with changes to routines (2, 3, 5, 13), many of which related to the sensory environment (7, 8, 12) and if adjustments were made, anxieties in the child often increased. For autistic children, this was identified as much more serious than for other children: “*the consequences are much more dire...and they leave a longer mark of anxiety...even if regular kids get anxiety ridden about the changes [when] they're tired and they're hungry...but with him it's like it build[s] up in his nervous system into this big mean anxiety blob”* (13). Yet families were also aware that long-term their child needed to build a capacity for flexibility in daily life, and the dangers of being too reliant on structured routines was a concern (3, 4). In many cases, parents had worked out contingency sensory strategies which assisted the child to adopt such flexibility to cope (9) and emphasizes the power of parents in steering their child's path.

### Theme 3: Orchestrating family life

The third theme of orchestrating family life captured positive sensory experiences for the child and doing family differently as subthemes. Due to the forensic sense making of sensory experiences, and forensic vigilance, many families had worked out which sensory sensitivities and preferences their child experienced most and could anticipate which family occupations were consequently most enjoyable. This theme relates to the orchestration of family life within the context of positive regulatory sensory experiences for the autistic child and doing family differently.

#### Positive sensory experiences for the child

Within this theme, parents described sensory occupations that their child appeared to enjoy and in general were perceived as positive experiences. For example, children were documented as enjoying the sensory experiences of a companion canine, which seemed to provide a calming influence on them: “*he's got a very calming effect on Eve… when she is feeling a bit down or anxious and then you know he's a bit of a comfort to her*” (9). Positive experiences within daily routines and sensory encounters were reported. For example, parents reported their child enjoyed bathing compared to other self-care routines which was attributed to the calming effect of warm water on the body (16): “*He likes to dance. He likes to dance around in circles, and then any time he is in the bathtub he is happy”* (6). Deep pressure tactile experiences were described for some of the children within the studies, with examples of children seeking out opportunities for close physical contact from parents (e.g., hugs, massage): “*We have special time watching [television] at home, we have family time. He likes sitting on the couch between me and his dad, the deep pressure cuddles”* (7). Parents were quick to point out this was different to other children: “*You can definitely tell that whenever you hug him it's not, um, it's not normal. He's definitely getting more out of it than just a hug”* (6).

Visual experiences were also evident in the studies, such as “*seeing everything*,” loving to see “*bubbles and balloons and things that fly around*,” and enjoying turning the light on and off (6). There were also other preferred experiences relating to the vestibular and proprioceptive senses which children sought out. “*He likes swinging, he loves being on a swing. Like when it was 25*°*F out and snowing, we were in the swing*” (6). “*We've got a swing that she can go and take herself on whenever she's feeling stressed out, the vestibular stimulation on the swing helps a lot to calm her down”* (7). “*He likes to jump. So, he jumps a lot, and he appears to get pleasure out of that”* (6).

Outdoor access to nature was identified as an important context as it provided opportunities for diverse sensory experiences that appealed to the autistic child, for example of natural objects including sticks or leaves (6, 8) or simply watching wildlife: “*our son is an avid bird watcher. He can sit for hours filming, photographing, and documenting them*” (8). Parents in another study also shared this view “*Going out on the boat and seeing the dolphins with the family makes her incredibly relaxed and happy. Anything with the wind in her face makes her very happy”* (7). Children in this study (7) enjoyed time spent with pets, walking, or biking around their neighborhood, and visiting the beach or the zoo. Parents and their children engaged in shared participation (7) more readily in high intensity sensory experiences such as swinging (6), hiking (6), fishing (5, 11), bike-riding (18).

#### Doing family differently

This theme relates to how families of autistic children function differently in the way in which families go about their daily occupations, rituals and routines when living with an autistic child. Doing family differently encompassed a range of experiences such as knowing every day is a different challenge in family routines, needing flexibility for the child, following their child's agenda, shared participation and going out together as a family. In a similar approach used by Goodman et al. (2007) in their study of “doing dress”, by naming this sub theme as “doing family”, the concept of family is expanded beyond simply considering what a family is (e.g., family members and where they live), to include ideas of meaning, agency and context within family relationships and occupations.

Within the studies, parents described how “e*very day is a different challenge*” (3). Being within the home environment appeared to provide a sense of safety, control and predictability within family routines (7, 8, 20). However, families described their days at home as being very busy and hectic (3, 4) with a significant part of the family's day revolving around the needs of the autistic child (4). Families described the differences in time pressures to get various routines fitted into the day such as eating, bathing and bedtime (3, 14). The morning routine for children and families was a key point raised (12), with getting the child up and ready in time for school being identified as a stressor in families: “*Getting him up for school in the morning is hard and you're encouraging, encouraging, encouraging him to get up, and he just gets angry … you know it's not going to be a good day in school*” (12).

Time spent in shared participation between parent and child typically pivoted around the child's occupational choices more than the parents' recreational preferences (11). Shared participation in activities as a family was usually dependent on whether the autistic child enjoyed those activities (3, 6, 11). Parents tried to be part of their children's activities and interests (2) and they would rather spend more of this time together (4). Parents found that the presence of a service dog in their family increased potential shared participation, on tasks such as grooming or petting the dog (2). Parents also reported that going places when their child had the support of their service dog such as ferry boat rides, airplane flights, weekends away were made possible (2).

Engaging in common family rituals such as going out together was discussed in some of the studies, yet due to the challenges of living with a child with sensory differences, families participated less often than they desired in activities such as shopping, going out to eat, family day trips, or vacationing (13). Going on a shopping trip could be a traumatic experience (13) and deciding to go someplace such as a restaurant or the cinema last minute was rarely an option as the child may not want to go inside once there. Given this context, there was a shared joy when families experienced success on these outings, for example being able to go to the supermarket and not have their child grabbing at things (4, 7)

Parents also noted other extra considerations that they put in place for example in outdoor nature: “*The unpredictability of wildlife! … We have deer in the yard, we have coyotes, there's bears*” (21). For these parents in Canada, their outdoor routines always required contingency plans for supervision of their child in these rural settings, to the extent that they had devised specific family safety plans to maximize success (21). Parents consequently sought safer, more enclosed outdoor places for leisure and play to avoid the constant need for supervision and to enhance the child's exposure to more independent movement outdoors.

Time together as a family was valued and prioritized (23): “*Stopping the rapid pace of life and having time together is appreciated. The more he spends time with his close family (like in family vacations), the bigger leap he makes.”* Some parents emphasized how family togetherness brought about positive shifts to the family dynamics. Their child's happiness was a core feature which parents reflected on. “*Like any other parent, it's happiness of course…you want your kids to reach milestones, reach independence”* (3).

## Discussion

This qualitative synthesis explored insights into parental perspectives of autistic children with sensory processing differences within the context of family life. Three core themes were identified and categorized as; (1) Sensory differences and routines in daily occupations, (2) What is hard about hard, and (3) Orchestrating family life. The studies within this review, all shared the lived experience from the perspective of parents on meaningful participation in daily occupations, and routines when living with an autistic child. To be successful in family occupations requires a complex integration of multiple elements including knowing what is hard about the hard, in order to navigate through daily life and orchestrate success. Success does not ignore what is hard- being vigilant and forensic in making sense of the child's experiences is fundamental to being able to enable occupational participation. Therefore, the challenges cannot be ignored but instead integrated and acknowledged so that challenges are inherent in understanding successful occupations. They co-exist.

This study explored sensory differences and routines in daily occupations in family life, because less is known about living a life of sensory differences and its relationship with constructing meaningful and successful shared family occupations. The synthesis of findings suggests that living a sensory life as an autistic child is made up of multi-sensory experiences that cannot be singularly siloed or individually categorized in many circumstances of daily life. Sensory processing differences were not reported in isolation (e.g., tactile hyperreactivity) or in sensory subtypes by the parents in the studies of this review, but were discussed as a part of daily occupations and family routines. Similar to Dickie et al. (2009), findings highlighted that a child's sensory differences are multifaceted, complex, fluid and embodied in occupations rather than being experienced in silos, as individual sensory processing issues. Nature, service dogs, participation in sports, engaging with playground equipment outside the home and physical touch from their parents such as hugging were reflected as being successful multisensory experiences for some autistic children. Aversive sensory experiences added another layer to the autistic child's participation challenges and consequently family participation. For example, mealtime participation highlighted the multisensory nature of a daily occupation which autistic children must contend with, whereby issues related to food were not limited to just one sensory aspect but included texture, taste, smell, visual aspects of the food itself, having food on their hands or tongue, alongside associated aspects such as predictability, routine, and novelty. This review exposes how sensory processing differences in autistic children impacts daily routines within the context of family life, which has been well reported within the literature (Kern et al., 2008; Schoen et al., 2009; Hochhauser and Engel-Yeger, 2010; Lane et al., 2010; Marco et al., 2011; Ismael et al., 2018). Findings from this review identified how family life must be adapted and changed to flow and function in accordance with the child's own sensory needs and preferences in the moment, but with the future child in mind. The adaption and change required for successful engagement in occupation, depended on parents' intense engagement with vigilance and forensic sense making to understand the child's sensory life.

What's hard about hard was significantly associated with the sensory emotional world. The sensory-emotional world experienced by the autistic child was very clearly depicted by parents throughout this review. Many of the associated emotions reported such as pain, distress, anxiety were linked back to the child's experience of sensory stimuli, and hence parents engaged in processes of forensic sensemaking to mitigate the negative influences of living a sensory life. Parents talked of the vigilance required to understand the child's lived experience, and through detective work understood that their child experienced sensation differently. This perception of the child's lived sensory experience has been illuminated in reviews of biographies for example in Conn's work, whereby autistic adults described the intense ecstasy and vivid memories of sensory experiences as children (Conn, 2015). Understanding the connection between emotional associations with sensory experiences enhanced a parent's ability to support their child in daily life and routines, yet not all parents in these studies had made the connection between the sensory-emotional world of their children. Further promotion of sensory awareness among families is warranted to maximize understanding to support meaningful participation.

Routines are often considered to be the epitome of stability, safety and security (Fiese and Parke, 2002). Routines make up the rhythm and fabric of family life and reflect how humans can form habits to enable participation in the environment (Clark et al., 2007). For example, family routines were often used by parents as a gateway to enhance the child's participation, e.g., going out together as a family, playing in the outdoors, sharing experiences in nature or in leisure activities their child preferred. However, in this review, it was evident that routines were often enacted as a necessity and for the most part, families often had no choice in what routines were completed because as noted above, they needed a strategy to mitigate and minimize the impact of the child's sensory and emotional experiences. In addition, it was evident that some family routines occurred that did not reflect the family values, for example, taking care of a child's personal hygiene and grooming through the use of restraint, or orchestrating different mealtimes for family members. This is one of the answers to the question what is “hard about hard”. Family routines may be adopted that reflect a mismatch between the values of the parents and the actions that they resort to using, which may be a result of lack of support, resources, education and/or societal pressures. Therefore, as health care professionals it is important that prejudice and judgment does not occur. An important consideration is that many think they know routines but unless you live a life with an autistic child, then the experience of routines can be very different. Hence, doing family differently needs to be accepted and embraced when addressing successful occupational participation in autistic children and their families.

Family-centered practice has been identified as a best practice framework when working with children with disabilities in health care internationally (Espe-Sherwint, 2008). The evidence base supporting this approach is strong (Dunst and Dempsey, 2007; Trivette et al., 2010) with effective outcomes for children and their families being reported in the literature (Dempsey and Keen, 2008). However, the use of this approach in practice should be continually reviewed and examined, to acknowledge the context and culture of the family narrative. One of the roles of occupational therapists within this area of practice is helping parents and families identify and understand how their child's sensory processing differences influence their daily occupations and participation in routines. Based on the findings of this review, more is needed to help parents to understand the links between sensory processing challenges and meaningful participation with the culture of family life specifically, and how supporting these functions, can champion family centered practice, in reality. Augmenting parents' understanding of how their children's sensory processing differences are linked to the specific daily routines and application of forensic sense making in their child's sensory processing is required in practice. This review builds on Boyd, Harkins-McCarthy and Sethi (2014) study, which prioritized the need for further research in this field to investigate how families successfully engage in shared daily occupations and routines within the context of family life across childhood and adolescence.

## Strengths and limitations

This paper is the first meta-ethnography to our knowledge that focuses on parental perspectives of autistic children and sensory processing differences in relation to meaningful participation in daily life, family routines and co-occupations and provides new interpretations of the subject matter. This paper was conducted in a robust manner ensuring high quality standards; with the authors adhering to the eMERGe and PRISMA guidelines. Greater diversity amongst the types of families included in the studies in this review, as well as having greater representation of families from more countries globally would allow for increased generalizability of findings. The context of many of the studies included in this review captured a minority world population. Interestingly when screening the studies, a high number from Asia and the Middle East focused on the stress associated with a autistic child's diagnosis, the parental burden of caregiving for an autistic child and the cultural stigmatism associated with such a disability. While efforts were made during the screening process to include studies across diverse cultures, it was notable that stigma in many studies was the more significant factor, rather than how to live family life successfully. No studies were found that addressed this topic of autistic children and their families using a strengths based perspective from majority world countries. Additionally, variance in the quality of articles reviewed may impact results reviewed in this study and this should be taken into consideration. For the majority of studies, the relationship between the researcher and participants had not been adequately considered and it was unclear in 6/23 studies whether the recruitment strategy was appropriate to the aims of the research. In 7 of the 23 studies, it was unclear whether ethical issues had been taken into consideration. A further limitation of this review is that strategies to support routines utilized by autistic children and families were not specifically focused on, as it was beyond the scope of this paper. Furthermore, one of the authors is a clinician working with families of autistic children and this personal bias may have influenced the results. This review was a collaboration between three white authors based residing within the same country. We acknowledge that while we represent some diversity of lived experience, including culture and education, our work is influenced by our relatively privileged backgrounds. We have all been raised in developed countries, and have all completed post-graduate university education.

## Future research

More research study of family routines and how this relates to a child's specific sensory differences is required, so a greater understanding exists of how to support a child's sensory needs in conjunction with their own and their families' daily occupations and routines. Future research needs to encompass a strengths-based approach to how autistic children with sensory processing differences engage in shared participation with their families. Further research within sensory processing and autism specifically, needs to focus on how family life gets addressed and how family values can be integrated into this intervention approach.

## Conclusion

This study reports on a meta-ethnographic synthesis that was conducted to illuminate the parental perspectives of autistic children within the family context. The findings of this study illuminated the sensory differences and routines in daily occupations, understanding on a deeper level what is hard about hard and the outcomes of orchestrating family life. As the prevalence of autism continues to rise and the demand for effective rehabilitation services increases for this population, a greater understanding is required on how families and their autistic children with sensory processing differences engage successfully in meaningful occupations, particularly within their own home environments and community settings.

## Data availability statement

The original contributions presented in the study are included in the article/Supplementary material, further inquiries can be directed to the corresponding authors.

## Author contributions

GD, HL, and JJ contributed to conception and design of the study, performed the qualitative analysis and synthesis, and wrote sections of the manuscript. GD and HL organized the database and wrote the first draft of the manuscript. All authors contributed to manuscript revision, read, and approved the submitted version.

## Funding

This work was supported by the Sensory Integration Network Limited (UK and Ireland), (Grant: PhD Research Projects Grant) as part of a PhD program in Occupational Science and Occupational Therapy, UCC, Cork, Ireland. The funder was not involved in the study design, collection, analysis, interpretation of data, the writing of this article, or the decision to submit it for publication.

## Conflict of interest

The authors declare that the research was conducted in the absence of any commercial or financial relationships that could be construed as a potential conflict of interest.

## Publisher's note

All claims expressed in this article are solely those of the authors and do not necessarily represent those of their affiliated organizations, or those of the publisher, the editors and the reviewers. Any product that may be evaluated in this article, or claim that may be made by its manufacturer, is not guaranteed or endorsed by the publisher.

## Footnotes

^1^Note: For this paper, the use of identity-first language (autistic child) will be applied. The preference of many autistic-led and autism-focused organizations, when talking about themselves and their condition, is to use autistic as their identity (Botha et al., 2021).

^2^Note the following numbers are used to represent the 23 articles included as eligible for this meta-ethnographic synthesis: 1, Burkett et al., 2021, 2, Burrows et al., 2008, 3, Columna et al., 2020, 4, DeGrace, 2004, 5, DeGrace et al., 2014, 6, Dickie et al., 2009, 7, Epstein et al., 2019, 8, Galbraith and Lancaster, 2020, 9, Harwood et al., 2019, 10, Keller et al., 2014, 11, Kim et al., 2018, 12, Kirkpatrick et al., 2019, 13, Larson, 2006, 14, Larson, 2010, 15, Marquenie et al., 2011, 16, Naik and Vajaratkar, 2019, 17, Potter, 2017, 18, Redquest et al., 2020, 19, Rios and Scharoun Benson, 2020, 20, Schaaf et al., 2011, 21, Shannon et al., 2021, 22, Suarez et al., 2014, 23, Tokatly Latzer et al., 2021.
