# Supplementary material for: Family life and autistic children with sensory processing differences: A qualitative evidence synthesis of occupational participation
Source: Front Psychol. 2022 Oct 20;13:940478. doi: 10.3389/fpsyg.2022.940478 (PMC9651035; doi:10.3389/fpsyg.2022.940478)
Supplement: Supplementary file 2 [file Data_Sheet_1.PDF]

To enable PROSPERO to focus on COVID-19 submissions, this registration record has undergone basic automated checks for eligibility and is published exactly as submitted. PROSPERO has never provided peer review, and usual checking by the PROSPERO team does not endorse content. Therefore, automatically published records should be treated as any other PROSPERO registration. Further detail is provided here.

Citation

Gina Daly, Helen Lynch, Jeanne Jackson. Family occupations and daily routines (FAIR) in children with autism spectrum disorder and sensory processing differences: a qualitative evidence synthesis.. PROSPERO 2022 CRD42022298938 Available from:
https://www.crd.york.ac.uk/prospero/display_record.php?ID=CRD42022298938

Review question

What is known about parental perspectives of children with autism spectrum disorder and sensory processing differences within the context of family life and routines?

Searches

The search strategy is being developed with the support of an academic support librarian in University College Cork.

A systematic search of peer-reviewed studies will be conducted using eight databases from health, science, education and humanities to ensure the inclusion of diverse perspectives: Academic Search Complete, CINAHL, ERIC, MEDLINE, PsycINFO, Scopus, Web of Science and PubMed.

Keywords used in the search will be drawn from recently conducted systematic reviews for children with autism spectrum disorder and from a review on strategy searching for qualitative research.

The search strategy will combine three concepts which are central to the research objective:

1. Autism Spectrum Disorder

2. Parental/caregiver perspectives

3. Qualitative research

The SPIDER search strategy tool will structure the process for screening and selection of studies.

Sample

Parents of children with a diagnosis of autism spectrum disorder aged between 3- 18 years old.

Phenomenon interest

Parental perspectives, views, experiences and opinions on their child’s participation in daily routines and family occupations is the central focus.

Design

Qualitative studies reporting primary qualitative data collection. If qualitative data from parents of children with autism spectrum disorder cannot be identified or is not sufficiently represented, the study will be excluded.

Evaluation

Qualitative analysis of parental experiences and perspectives of living with and supporting a child with autism spectrum disorder. Studies where a method of qualitative analysis is not described, or where an in-depth analysis and exploration of parental views is not clear will be excluded. This must be represented through the first order quotes and second order categories presented in the findings of the paper.

Research Type

• Peer reviewed journal articles

• Full text available in English

• Studies published between the years 2000 -2021

• Systematic reviews, protocols, theoretical work, editorials, opinion pieces and dissertations will be excluded

Types of study to be included

All qualitative studies reporting qualitative data collection of perspectives, experiences, feelings, views and opinions of parents of children with autism spectrum disorder (e.g., data collected through qualitative methods such as interviews, focus groups, or participant observation etc.) will be included. Studies must describe how qualitative analysis was completed.

Studies where a method of qualitative analysis is not described, will be excluded. Studies where only quantitative methods were used will be excluded.

Condition or domain being studied

Autism spectrum disorder in children is the condition being studied.

Diagnostic manuals, ICD-10 and DSM-5, set out the criteria for autism spectrum disorder to be diagnosed.

Participants/population

Inclusion: Parents of children with a diagnosis of Autism Spectrum Disorder (as diagnosed using any recognised diagnostic criteria) aged between 3-18 years old. Parents of children with a diagnosis of autism spectrum disorder and a co-occurring condition such as an intellectual disability, sensory processing disorder and/or another neurodevelopmental condition such as ADHD, dyspraxia, dyslexia, GI problems, OCD and other mental health difficulties will also be included as there is a high co-occurrence rate in autism with these conditions.

Exclusion: Parents of children who have a diagnosis of autism spectrum disorder outside the age range of 3-18 years and have a co-occurring physical disability will be excluded. Parents of children who do not have a diagnosis of autism spectrum disorder will be excluded.

Intervention(s), exposure(s)

Inclusion: Studies will be included if they report parents descriptions of their views, perspectives and opinions on their child’s participation in daily routines and family occupations.

Exclusion: Studies will be excluded if the primary focus is not on the child's daily routines and participation in family occupations but is on for parental distress, coping with the burden of a diagnosis of autism spectrum disorder, trauma or marital relationships for example.

Comparator(s)/control
Not applicable

Context
Inclusion: All cultural and geographic contexts will be considered. Settings such as home and the community where the parent is present with the child will be included.

Excluded: School, airports, hospitals and dental surgeries where the parent is not present with their child and where it does not represent typical family life and daily routines will be excluded.

Main outcome(s)
The review aims to:

1. Systematically review and synthesise qualitative literature regarding parental/caregiver perspectives of children with autism spectrum disorder and atypical sensory processing patterns on their occupational participation in daily routines and family occupations.

2. Direct health service delivery and the development and implementation of services for families with children of autism spectrum disorder whereby family experiences of daily life and occupations informs occupational therapy practice.

Measures of effect

Not applicable

Additional outcome(s)
Not applicable

Measures of effect

Not applicable

Data extraction (selection and coding)
All retrieved papers will be imported into Endnote reference management software, and duplicates will be removed. Next, the remaining citations will be uploaded to Covidence software which is designed to facilitate independent screening when completing a review. Two reviewers will screen all study titles and abstracts against the inclusion and exclusion criteria. In this way, all titles and abstracts will be screened twice.

Two reviewers will conduct the full-text review for all included papers. Where discrepancies arise in the inclusion of papers for full text review, the third reviewer will help to facilitate a final decision. Any disagreements will be managed through discussion until a consensus is reached.

All included studies will be read in full to ensure they meet all inclusion criteria by two reviewers of the team. The reference lists of all included studies will be reviewed to identify potential further studies for inclusion.

Data on the characteristics of included studies will be extracted from the included full-text studies and entered into a customised excel datasheet by one reviewer and checked for accuracy by another. The data extraction form will include the categories such as; citation, study setting/country, sample size, participant characteristics, aims of the study, methodology (design, data collection and data analysis), findings and outcomes.

First and second order constructs (participant quotes and author interpretations) will be extracted from all included papers and imported into Nvivo software for analysis.

Risk of bias (quality) assessment
In this review, one quality assessment tool will be used; the Critical Appraisal Skills Programme (CASP) qualitative studies checklist will be completed on each of the papers selected for the final review (https://casp-

uk.b-cdn.net/wp-content/uploads/2018/03/CASP-Qualitative-Checklist-2018_fillable_form.pdf). All papers that meet the inclusion criteria will be subjected to appraisal using the CASP checklist by two independent reviewers. All studies will be critically appraised and any differences of opinions will be resolved by discussion and the involvement of a third reviewer if necessary.

Strategy for data synthesis

Meta-ethnography will be used to synthesise and evaluate the results of the included studies. Noblit and Hare’s (1988) seven step approach for conducting analysis in meta-ethnography will be used. The stages include getting started, deciding what is relevant to the initial interest, reading the studies, determining how the studies are related, translating the studies into one another, synthesising the translations and expressing the synthesis.

Each of the included full-text studies will be imported into QSR Nvivo qualitative data analysis software to facilitate extraction of second-order concepts, coding and comparison. As suggested, by Noblit and Hare (1988) all studies will be read several times in full. Key quotations, metaphors, and concepts related to parental perspectives of daily routines and family occupations in children with autism will be extracted using the words and explanations provided by the authors (second-order constructs). Throughout, the process of meta-ethnographic analysis and synthesis, two reviewers will initially complete coding and data extraction independently and will then meet to collaborate and compare regarding their findings. Further syntheses will occur regarding emerging themes.

Analysis of subgroups or subsets

A sub-group analysis may be completed if the data permits for example; age range of children, cultural contexts and/or community versus home settings. This may be significant to consider within the context of family centred practice to avoid a universal approach in practice to any findings identified.

Contact details for further information

Gina Daly
107567626@umail.ucc.ie

Organisational affiliation of the review

University College Cork
www.ucc.ie

Review team members and their organisational affiliations

Ms Gina Daly. University College Cork, Cork, Ireland
Dr Helen Lynch. Department of Occupational Science & Occupational Therapy, Brookfield Health Sciences Complex, University College Cork, Cork. Ireland.
Professor Jeanne Jackson. Department of Occupational Science & Occupational Therapy, Brookfield Health Sciences Complex, University College Cork, Cork. Ireland.

Type and method of review

Synthesis of qualitative studies, Systematic review, Other

Anticipated or actual start date

01 January 2022

Anticipated completion date

14 February 2022

Funding sources/sponsors

Funding Sources:

Gina Daly is a PhD Student in the Department of Occupational Science & Occupational Therapy, Brookfield Health Sciences Complex, University College Cork, Cork. Ireland. Grant Award funding has been received by Sensory Integration Education UK & Ireland and the Elisabeth Casson Trust UK throughout the PhD programme.

Sensory Integration Education UK & Ireland (SI Network research grant): https://www.sensoryintegrationeducation.com/

The Elizabeth Casson Trust UK (Doctoral Studies Grant Award): https://elizabethcasson.org.uk/support-development/funding/doctoral-studies/

Grant number(s)

State the funder, grant or award number and the date of award

Funder: Sensory Integration Education UK & Ireland

Grant award number: Not applicable

Date of Award: 26/05/2018

Funder: The Elizabeth Casson Trust UK

Grant award number: Not applicable

Date of Award: 27/09/2018 & 15/07/2020

Conflicts of interest

Language

English

Country

Ireland

Stage of review

Review Ongoing

Subject index terms status

Subject indexing assigned by CRD

Subject index terms

MeSH headings have not been applied to this record

Date of registration in PROSPERO

17 January 2022

Date of first submission

17 December 2021

Details of any existing review of the same topic by the same authors

Not application

Stage of review at time of this submission

Stage	Started	Completed
Preliminary searches	Yes	No
Piloting of the study selection process	No	No
Formal screening of search results against eligibility criteria	No	No
Data extraction	No	No
Risk of bias (quality) assessment	No	No
Data analysis	No	No

The record owner confirms that the information they have supplied for this submission is accurate and complete and they understand that deliberate provision of inaccurate information or omission of data may be construed as scientific misconduct.

The record owner confirms that they will update the status of the review when it is completed and will add publication details in due course.

Versions

17 January 2022

17 January 2022
